# Computer-aided assessment of diagnostic images for epidemiological research

**DOI:** 10.1186/1471-2288-9-74

**Published:** 2009-11-11

**Authors:** Alison G Abraham, Donald D Duncan, Stephen J Gange, Sheila West

**Affiliations:** 1 Epidemiology Department, Johns Hopkins Bloomberg School of Public Health, Baltimore, Maryland, USA; 2 OGI School of Science and Engineering, Oregon Health and Science University, Beaverton, Oregon, USA; 3 Wilmer Eye Institute, Johns Hopkins Hospital, Baltimore, Maryland, USA

## Abstract

**Background:**

Diagnostic images are often assessed for clinical outcomes using subjective methods, which are limited by the skill of the reviewer. Computer-aided diagnosis (CAD) algorithms that assist reviewers in their decisions concerning outcomes have been developed to increase sensitivity and specificity in the clinical setting. However, these systems have not been well utilized in research settings to improve the measurement of clinical endpoints. Reductions in bias through their use could have important implications for etiologic research.

**Methods:**

Using the example of cortical cataract detection, we developed an algorithm for assisting a reviewer in evaluating digital images for the presence and severity of lesions. Available image processing and statistical methods that were easily implementable were used as the basis for the CAD algorithm. The performance of the system was compared to the subjective assessment of five reviewers using 60 simulated images. Cortical cataract severity scores from 0 to 16 were assigned to the images by the reviewers and the CAD system, with each image assessed twice to obtain a measure of variability. Image characteristics that affected reviewer bias were also assessed by systematically varying the appearance of the simulated images.

**Results:**

The algorithm yielded severity scores with smaller bias on images where cataract severity was mild to moderate (approximately ≤ 6/16^*ths*^). On high severity images, the bias of the CAD system exceeded that of the reviewers. The variability of the CAD system was zero on repeated images but ranged from 0.48 to 1.22 for the reviewers. The direction and magnitude of the bias exhibited by the reviewers was a function of the number of cataract opacities, the shape and the contrast of the lesions in the simulated images.

**Conclusion:**

CAD systems are feasible to implement with available software and can be valuable when medical images contain exposure or outcome information for etiologic research. Our results indicate that such systems have the potential to decrease bias and discriminate very small changes in disease severity. Simulated images are a tool that can be used to assess performance of a CAD system when a gold standard is not available.

## Background

Diagnostics are becoming increasingly image based. Whether the setting is clinical practice or research, information must be extracted from an image to determine disease status. The determination of the presence or severity of disease will impact clinical care for a patient or outcome status in a study. In many clinical arenas images are assessed using subjective methods that depend upon the skill and consistency of a reviewer. The performance of screening mammography has been shown to be highly dependent upon the reader's skill and training [1,2]. The use of computer-aided diagnosis (CAD) systems to improve the sensitivity and specificity of lesion detection have become a focus of medical imaging and diagnostic radiology research [3]. Such systems have been explored extensively as a method for improving the detection of breast cancers from mammography [4,5] and the evidence indicates CAD can improve the accuracy of detection [6]. These CAD systems have also been employed in lung cancer and other tumor diagnosis. Evaluation of such systems can be challenging since the quality of the images, the application and expertise of the user will all contribute to the detection performance. Established methods such as receiver operating characteristic (ROC) analysis and free-response receiver operating characteristic (FROC) analysis can provide metrics for assessing performance given knowledge of the true disease classification. Such methods are not easily adapted, however, to assessing performance when the outcome is polytomous or continuous, though methods have been explored for handling multi-class and continuous measurements [7-11]. Another consideration is the ascertainment of the true disease status. Biopsy can provide a gold standard (true tumor presence) for cancer diagnostics but simple gold standards for other image diagnostics or for outcomes other than presence of disease (e.g. disease progression) may be challenging to find.

Perhaps as a result of a limited ability to assess performance when ROC analysis isn't practical, CAD systems have primarily been used clinically to locate lesions such as breast tumors. However, their application could be extended to the research setting. Disease incidence is a primary outcome in epidemiologic studies. Further CAD systems could be adapted to outcomes other than the presence or absence of disease. Progression and severity are disease outcomes of interest that can be assessed in diagnostic images. Regardless of the outcome, minimizing measurement error is important for making valid inferences and CAD systems have the potential to reduce bias and misclassification in other applications besides tumor detection. An additional advantage is the ability to calibrate the detection algorithm to adjust the balance between false positives and false negatives to incorporate the cost of missing true cases or falsely identifying non-cases.

Using the example of cortical cataract detection, we developed a software algorithm for aiding in the evaluation of digital images for the presence and severity of lesions. The CAD system was designed 1) to assist a subjective reviewer in identifying lesions in a lens image and assigning a severity score and 2) to use accessible statistical and image processing methods that could be readily implemented through available software. Standard assessment of lens images is done using semi-qualitative classification schemes involving a trained reviewer and a standardized scale of severity [12-18]. Stand-alone software algorithms have been used previously to attempt to improve cataract severity measurement and have shown reasonable agreement with standard reviewer-based methods but were limited in their application and subsequent use [19-27]. We hypothesized that a CAD system that assisted a trained reviewer could reduce measurement error and would be feasible to implement with standard software.

Since the true severity of cortical cataract is unknown, we assessed the performance of the CAD algorithm using simulated images with known severity created to mimic the characteristic appearance of diagnostic lens images. Cataract is a disease processes that alters the structure of the lens to degrade lens transparency. Cataract severity is primarily of interest in the research setting as an outcome for studying risk factor associations and, potentially, for evaluating treatments or interventions. Clinically, an assessment of vision and patient perception of vision difficulty are the metrics used to indicate for cataract surgery. Thus cataract severity does not solely determine the occurrence of surgery; individual and physician factors contribute as well. However, epidemiolgic research, based on an assessment of cataract severity, is important given the high prevalence in older age groups, estimated to be 54.2% among African Americans and 24.2% among Caucasians [28]. Improving upon cataract severity measurement using computerized assessment methods could provide a means for cataract researchers to explore more subtle risk factors associated with disease progression. The ability to develop a CAD system that minimized measurement error using available software packages would, in general, indicate the feasibility of wider application of CAD in research settings.

## Methods

**Figure 1:**
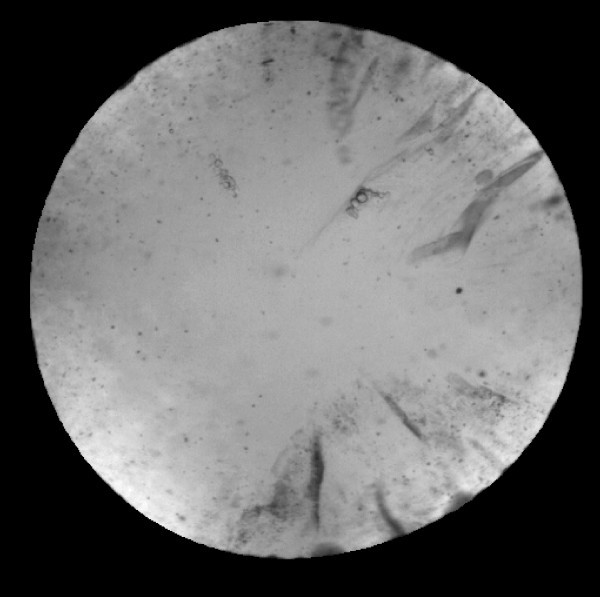
**Imaging the cortical surface of the lens**. Retroillumination images capture the backscatter of light reflected off the back of the eye. The darker areas in the image correspond to cortical opacities that reduce the clarity of the lens and result in less light returning to the camera. The grey level intensity values range from 0 to 255.

Cortical cataracts are assessed using retroillumination images that capture the backscatter of light reflected off the back of the eye. Areas of the lens surface that have reduced clarity (such as those with cortical opacity) will appear darker in the image, the result of less light returning to the camera. These images are captured through the dilated pupil resulting in a circular area of interest with cataract opacity information. The remainder of the image outside the pupillary margin is dark. Pixels, the informational building blocks of a digital image, are the unit of observation, resulting in very large datasets. A standard resolution for digital retroillumination images yields a dataset of 512 by 512 pixels or 262,144 observations with light intensity often represented as 8-bit integer values from 0 (black) to 255 (white)(Figure 1). Digital retroillumination images from the Salisbury Eye Evaluation (SEE) Study [29] were used to train the CAD system and parameter values were determined empirically based on performance in identifying opacities (as assessed by a trained reviewer) in the SEE dataset of 601 right eye lens images and 603 left eye lens images. Identifying cortical opacities in real image data required an algorithm that could 1) standardize images for lighting inconsistencies across the image, 2) find the region of the image that contained opacity information (the lens) and 3) categorize each pixel as diseased or normal in order to obtain a final area of disease coverage (i.e. a final severity score from 0.0 to 16.0). For the purposes of the current study, only the first and third steps were required since the boundary of the pupil was known. However, we describe the methods for all three steps in the following sections:

### Image processing

Eliminating noise, when feasible, improves measurement in general and, for CAD algorithms, increases the performance consistency. Retroillumination images are taken using cross-polarized light to reduce the light reflex artifact in the images. The result is light intensity heterogeneity across an image that can amplify or attenuate the appearance of cortical cataract opacities. Thus a filtering or image processing step is needed prior to attempting to identify cataract opacities. If the distribution of light across the image were known, an image could be standardized to remove the effect of background intensity on an estimate of opacity severity. We estimated the background intensity, *B*, by averaging intensity information locally using mathematical morphologic procedures called erosion/dilation operations [30]. Implementation was accomplished using the Matlab Image Processing Toolbox (The Mathworks, Inc) imdilate and imerode functions with an ellipsoid structuring element. Dividing the original image, *M*, by the result, *B*, yields an image with a standardized background lighting.

### Segmentation

Often images contain structures or areas that are not of interest. For example we could rule out ribs in a chest X-ray when assessing for lung tumors. Thus if we can define the boarders of the regions of interest (segment the image) we can often simplify the decision or classification rules necessary for separating normal from abnormal. The pupillary margin bounds the area within a retroillumination image that contains cataract severity information so segmentation is used to eliminate the pixels outside the margin that contribute no information about opacity. We need to identify this boundary, which is equivalent to estimating the function that describes the boundary shape and placement in the image. For this application, we used a specialized edge detector called a deformable contour model, which can find irregularly shaped contours. First formulated by Witkin et al. [31] and improved by Cohen [32], deformable contour models are constrained splines that can be used in a variety of image applications. The contour models were implemented by adapting Matlab code available from the work of Xu and Prince [33].

### Classification

Decision thresholds are used with any surrogate measure of disease to define the subgroup who will receive intervention, treatment, further diagnostics, or be considered to have the outcome for the purpose of epidemiologic research. The goal is to minimize the percent of false positives and false negatives, which is challenging as diseased and non-diseased individuals have distributions of values of the surrogate measure that often overlap. Defining cortical cataract for each observation (pixel) in a retroillumination image is based on the surrogate measure of grey level. The grey level values in a retroillumination image are a function of the external illumination and opaqueness due to disease. After compensating for the variation in light intensity across the image we assume that only the degree of disease in the standardized image determines the grey level. To discriminate between diseased (dark) and non-diseased (light) pixels we used fuzzy c-means clustering [34,35]. Fuzzy clustering is a method of classification that allows membership in a cluster to be partial. For each pixel observation *i *= 1... *N*, the degree of membership in the diseased and non-diseased clusters was estimated through minimization of a function that describes the cluster criteria and how proximate each pixel is to the criteria. Implementation was accomplished by defining the membership functions and iterating to obtain the degree of membership for each pixel. Final classification was taken as the cluster (cataract or normal) for which a pixel had the highest degree of membership. The methods described above yielded a CAD algorithm for cortical cataract that suggested to the user which pixels were cataractous and provided an estimate of the percent of the total viewable lens area covered by cataract, a standard measure of cortical cataract severity. This severity score was a continuous measure and was normalized to the scale of 0.0 to 16.0 to mimic standard grading methods. The implementation was done in Matlab version 7.0.4 (Mathworks Inc) and an interface was built in LabView 7.1 (National Instruments).

### Validation

The validity of reviewer-based or computer-based cataract severity measurement has never been assessed since no gold standard exists. Simulated data are drawn from known distributions such that the true disease status is known. Thus simulation studies are an inexpensive way to obtain an estimate of validity, albeit in an idealized setting. For evaluating a CAD algorithm, simulation studies are easily implemented. Digital images can be created that capture various aspects and stages of the lesion of interest. Noise and artifact may be added to challenge the system or all noise can be eliminated to test the optimal performance.

**Figure 2:**
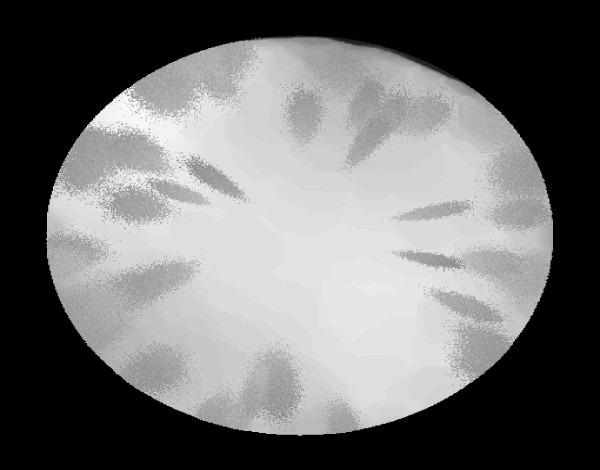
**Simulated lens image**. An example of a simulated retroillumination image created by drawing clusters of pixels from a trivariate normal distribution and placing them on an non-diseased background image.

For our purposes we chose to model retroillumination images by assuming each area of cortical opacity in an image, *M*, is a cluster of pixels randomly drawn from a trivariate normal distribution. Simulated retroillumination images were created by placing clusters of 5000 opacity pixels drawn from trivariate normal distributions on a standard background image with a fixed pupillary margin (thus the segmentation step in the CAD algorithm was not tested) and heterogeneous light intensity across the image. No additional artifacts were added to the image. Parameter values were determined empirically to mimic the appearance of real data. A gamma distribution was used to describe the placement of the opacities across the lens, which tend to be increasingly prevalent along the perimeter of the lens. An example of the resulting simulated images is shown in Figure 2.

To test the dependence of the CAD performance on image characteristics, the appearance of the images was systematically altered by varying the number of opacities (5 levels), the contrast between diseased and non-diseased areas (3 levels), the width of opacities (2 levels) and the length of the opacities (2 levels). This resulted in 60 simulated images for assessment. The images were each assessed twice by the CAD algorithm and separately by five trained reviewers using a standard assessment method, the Wilmer Eye Institute cortical cataract classification system [13]. The Wilmer classification system uses a seventeen category severity scale with possible scores ranging from zero to sixteen. Reviewers were told to identify all cortical opacities in the image and estimate the area they cover in 16^*ths*^. A circle divided into sixteen pie-shaped wedges is overlaid on the images to provide a visual guide for estimating the area involved. To standardize the assessment, a training set of retroillumination images was presented to all the reviewers. Consensus was achieved to within one severity unit on all training images.

Using language R, between- and within-reviewer variability was estimated. The bias between the mean estimated severity assigned by each reviewer and the true severity was determined and the agreement between the CAD algorithm and the reviewers was assessed. A mixed-effects model was used to examine the effect of each parameter and the choice of method (reviewer or CAD) on the bias between the estimate and the truth.

## Results

**Figure 3:**
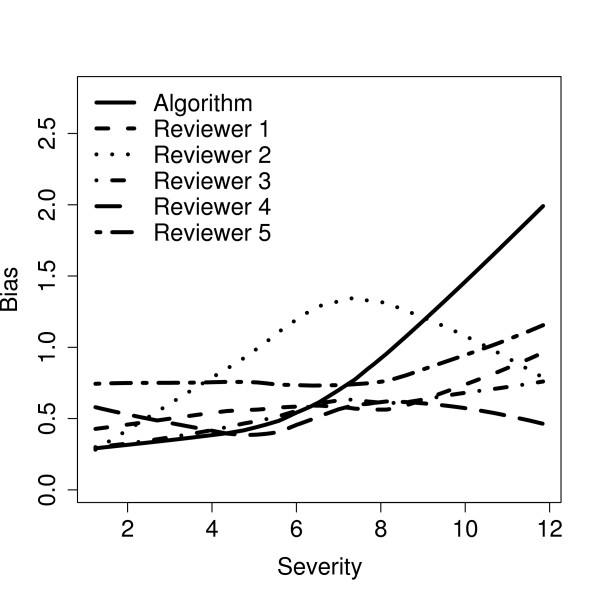
**Plot of bias versus true severity**. The bias between the average severity assigned to an image and the true severity plotted versus the true severity. Locally weighted least squares was used to capture the trend in the data. The range of true severity in the simulated images was 1.2 to 11.8 out of a possible range of 0.0 to 16.0.

Both the reviewer and the CAD system reported a severity score from 0.0 to 16.0. The absolute difference between the mean severity given to each image and the true severity (bias) indicated that the CAD system outperformed the reviewers when the opacity was relatively mild (Figure 3). The bias associated with the CAD system increased as the severity increased and we found the bias was consistently in the direction of underestimating the image severity. For the reviewers, the correlation between absolute bias and severity was more moderate and the average bias remained between 0.54 and 1.13 grade units. Comparing all reviewers to the CAD system, the average bias was the same at 0.77 severity units as seen in Table 1. Using ROC analysis and assigning a score of 3.0 (approximately the point at which vision becomes noticeably affected in vision tests) as the threshold for categorizing an individual as having cataract, the area under the curve was greater than 0.98 for all reviewers and the CAD system, providing little discrimination between methods. When agreement between the reviewers and the CAD system was evaluated, we found the mean severity from each reviewer tended to be higher than that estimated by the CAD algorithm. On average, the difference between the methods increased with increasing severity. The trend mirrored that of the bias seen in Figure 3.

**Table 1:** Bias and variance.

Assessment	Bias	Variance
Reviewer 1	0.64	0.76
Reviewer 2	1.13	1.22
Reviewer 3	0.54	0.56
Reviewer 4	0.68	0.98
Reviewer 5	0.87	0.48
Average of Reviewer	0.77	0.80
CAD Algorithm	0.77	0.00

CAD - Computer-aided diagnosis

The average bias and variance of the reviewer and CAD algorithm marginal across cataract severity.

The parameter estimates for the fixed elements of the mixed model produced the values for mean bias seen in Table 2. The reviewer had a small, non-significant increase in bias compared to the CAD system when looking marginally across severity and controlling for various image features. When there were few opacities on an image, reviewers typically over-estimated the severity. The bias reversed direction as the number of opacities increased. Bias was minimized when opacities were darker in appearance (increased contrast) and when opacities were relatively wide and short (more circular in appearance). In comparison, long, narrow opacities tended to promote overestimation of the severity. The estimated parameter for the random component of the intercept for the CAD system indicated that it had very low bias when the severity was low, the opacity contrast was low, and the opacities were small and round. Under those conditions, the CAD algorithm had the smallest bias, on average. However, as the number of opacities in an image increased, the CAD system tended to underestimate the severity more than the average performance, as seen from the fixed-effects estimates.

**Table 2:** Predictors of reviewer bias.

Variable	Fixed parameter estimate*	Standard error	*P*
Intercept	0.25	0.31	0.417
More opacities	-0.21	0.08	0.005
Darker opacities	-0.07	0.10	0.472
Wide opacities	-0.17	0.10	0.075
Long opacities	0.31	0.10	0.002
Reviewer	0.04	0.27	0.879

*Bias = estimated severity - true severity

In cataract severity score units

Parameter estimates for the fixed-effects from the linear mixed effects model analysis of image factors affecting bias.

Multiple reviewers provided an estimate of the between- and within-reviewer variability. The between-reviewer variability tended to increase with increasing opacity severity while the within-reviewer variability did not show a consistent trend. The variance marginal across severity for the reviewers ranged from 0.48 to 1.22 with an average of 0.80. The CAD system in isolation had no variability, as it processed the same image identically each time. Thus, the variability is dependent upon the reviewer using the CAD system.

## Discussion

As diagnostic imaging has become more widely used in the clinical setting, the opportunity for images to be a source for outcome and exposure assessment in epidemiologic research is growing. Assessment by a clinician or trained reviewer is one standard diagnostic methodology for making a disease determination from such images. Computer-aided diagnosis systems were introduced to facilitate this task. In this article we detailed how a CAD system can be developed for research purposes when medical images contain valuable exposure or outcome information for answering a research question. In our example in the field of opthalmologic epidemiology, a CAD system for assessing cortical cataract severity from retroillumination images was designed using available and established image processing and statistical techniques. We further examined the utility of using simulated images to validate image-based measurement or assessment in the absence of a gold standard. From our simulation study we found that the CAD algorithm outperformed the trained reviewer in estimating cataract severity from images with mild to moderate cataract involvement. The reduction in bias observed with the CAD system likely reflects both improved performance at assessing severity as well as the ability of the system to describe severity on a continuous scale. The coarsening of the data through the use of a categorical scale limits how close the reviewers can be, on average, to the true severity. When the true disease status was dichotomized for the ROC analysis, we found that the performance of the reviewers was equivalent to the CAD system, suggesting that, in this example, most of the improvement arises from the CAD algorithm's ability to discriminate very small changes in severity. On more severe cases, the CAD system had higher bias due to problems in the background noise filtering methods. Replacing the erosion/dilation operations with a more robust method of adjustment for uneven background lighting would likely result in an assessment algorithm with low bias at all severities. Methods are available for improving upon background intensity standardization in images [36-39]. We chose erosion/dilation operations for their ease of implementation with standard software. It should be noted that severe cases of cortical cataract are rare since cataract surgery is often performed before the cataract progresses to such an extent.

A feature of the CAD system worth highlighting is the zero variability. The suggested areas of opacity will not vary with repeated assessment of the same image. The reviewer using the CAD system may be more or less adherent to the suggestions of the system, which, we hypothesize, would increase the variability to a maximum that would be the variability of the reviewer making unassisted decisions about severity. Therefore, we would expect that the algorithm assisted reviewer would have, on average lower within-reviewer variability. While we did not evaluate the impact of the CAD system on reviewers' performance, this is an important question that would need evaluation prior to implementation. Differences in the effect of CAD on a reviewer's assessment of an image would best be evaluated using real image data, where image interpretation would be most challenging and results would represent performance in practice. It is clear that trained reviewers are sensitive to various aspects of the images or lesions and this could result in biases that vary from study to study. Reviewers were sensitive to the contrast and performed better with certain opacity shape characteristics, which may have implications for cataract research using standard severity assessment methods. Cortical cataract opacities tend to take on a variety of appearances and, to the extent that the shape may be related to the mechanism, studies of some risk factors may be more prone to bias, potentially differential.

There are numerous aspects of reviewer behavior and performance that could be studied using simulated images. It is difficult to assure that the assessment process of a reviewer would be the same with simulated versus real images. A simulation study could not stand in isolation as the only evaluation of an assessment method and are only valuable when knowledge of true disease status cannot be attained. However, simulation studies are low cost, do not impact patients, and allow for a fuller exploration of the strengths and weaknesses of the assessment method.

## Conclusion

Subjective reviewers of lens images can accomplish complex discrimination tasks that cannot be fully automated at present. However, we found that the performance of reviewers is affected by various features in the lens image and the degree of bias in their assessment may vary from image to image. Augmenting the assessment process with a computer algorithm is a means of standardizing the measurement and minimizing some of the variability of subjective image assessment. Such CAD systems can be designed for many different applications with readily available image processing and statistical software. Testing and validation can readily be performed using simulated images that capture the main features of interest.

## Competing interests

The authors declare that they have no competing interests.

## Authors' contributions

AA provided most of the coding and analysis for this project along with writing the bulk of the manuscript. DD was the technical adviser for choosing appropriate image processing and analysis tools to develop the lens image assessment algorithm. DD was formerly at the Applied Physics Laboratory of Johns Hopkins and contributed to multiple projects at the Wilmer Eye Institute that required engineering expertise and image analysis. SG is a biostatistician and provided assistance with statistical methods and the creation of the simulated images. SW provided image data from the Salisbury Eye Evaluation Study for initial testing and pilot studies related to this project. SW also provided expertise with lens grading and training for the lens graders who contributed to the study. All authors read and approved the final manuscript.

## Pre-publication history

The pre-publication history for this paper can be accessed here:

http://www.biomedcentral.com/1471-2288/9/74/prepub
